# Antifouling Activity of Simple Synthetic Diterpenoids against Larvae of the Barnacle *Balanus albicostatus* Pilsbry

**DOI:** 10.3390/molecules15118072

**Published:** 2010-11-09

**Authors:** Jun-De Chen, Rui-Zao Yi, Dan-Qing Feng, Yi-Ming Lin

**Affiliations:** 1 Research Center for the Chemistry and Chemical Engineering of Marine Biological Resource, The Third Institute of Oceanography of the State Oceanic Administration, Xiamen 361005, China; 2 Department of Biology, School of Life Sciences, Xiamen University, Xiamen 361005, China; 3 Department of Natural Products Chemistry, Institute for Biomedical Research, Xiamen University, Xiamen 361005, China; 4 Department of Oceanography, College of Oceanography and Environmental Science, Xiamen University, Xiamen 361005, China

**Keywords:** pimarane diterpenoid, synthesis, antifouling activity, structure-activity relationship

## Abstract

Five new pimarane diterpenoids **1-5 **were synthesized using ent-8(14)-pimarene-15R,16-diol as starting material. The structures were elucidated by means of extensive NMR and MS analysis. The antifouling activity against larval settlement of the barnacle *Balanus albicostatus* were evaluated using capsaicin as a positive control. Compounds **1-3 **and **5 **showed more potent antifouling activity than capsaicin. Compound **5**, which exhibited almost the same antifouling activity as starting material, showed better stability than starting material. These compounds all showed antifouling activity in a non-toxic way against larval settlement of the barnacle *B. albicostatus*. Analysis of structure-activity relationships (SAR) demonstrated that the substituents on the C-15 and C-16 position of pimarane diterpenoid were responsible for the antifouling activity.

## 1. Introduction

It is well known that marine fouling organisms often cause technical and economic problems by settling on artificial surfaces submerged in seawaters [1]. Although antifouling paints containing organotin compounds such as tributyltin (TBT) and tributyltin oxide (TBTO) are very effective for controlling these fouling organisms, they have been found to be toxic to many non-target marine organisms [2], and The Marine Environment Protection Committee of the International Maritime Organization has proposed a ban on the application of TBT-based antifouling paints as of January 1, 2008 [3]. Currently, some booster biocides have been used in antifouling paint formulations, but they may also pollute the aquatic environments [4], thus there is an urgent demand for new antifouling agents which are both effective and environmentally friendly. A significant source of effective and environmentally friendly natural antifoulants, *i.e.,* terpenoids, steroids, fatty acids, amino acids, heterocyclics, acetogenins, alkaloids, and polyphenolics, etc. have been reported [5,6,7,8,9,10]. Moreover, a series of terpenes have been synthesized and evaluated for antifouling activity against the larvae of the barnacle [11,12,13,14,15]. In the course of our investigations on environmentally friendly antifoulants, we observed that pimarane diterpenoids can exhibit non-toxic antifouling activities against larval settlement of the barnacle *B. albicostatus* [10]. The structure-activity relationship (SAR) analysis of these pimarane diterpenoids suggested that the antifouling activity might be due to the hydroxyl group on the C-15 and C-16 position of these compounds [10]. In order to further investigate the effect of free hydroxyl group and substituents on the C-15 and C-16 position of pimarane diterpenoid on the antifouling activity, we used ent-8(14)-pimarene-15R,16-diol as the starting material and then synthesized five new pimarane diterpenoids **1**-**5**. These compounds were tested for their antifouling activity against larval settlement of the barnacle *Balanus albicostatus*, which is an important fouling organism in East Asian coastal waters. Capsaicin was used as a positive control to evaluate the antifouling activity of the synthetic compounds in our experiment because it was widely used for controlling fouling organisms in a non-toxic way around the World [16,17,18]. Moreover, the SAR of these compounds was also discussed.

## 2. Results and Discussion

### 2.1. Chemistry

**Scheme 1:**
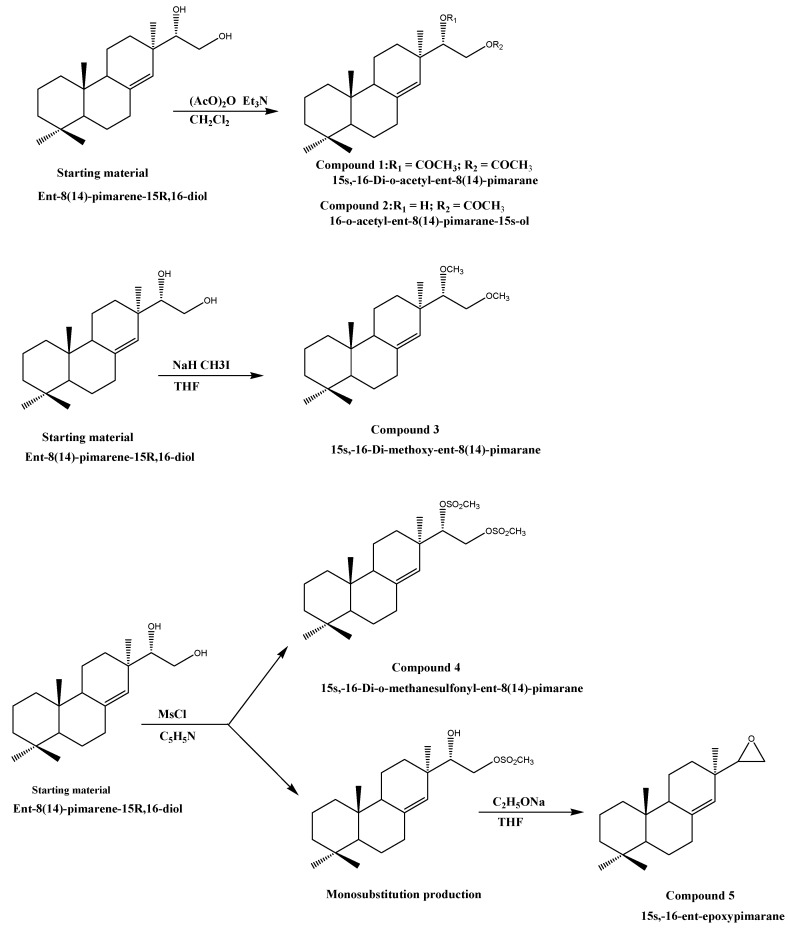
Synthesis of the investigated diterpenoids.

Ent-8(14)-pimarene-15*R*,16-diol was used as starting material due to its potent antifouling activity with EC_50_ value of 0.04 µg/cm^2^ [10]. Compounds **1** and **2** were synthesized from the starting material by an acylation reaction with acetic anhydride in the presence of triethylamine. Alkylation of the starting material with methyl iodide in the presence of sodium hydride and tetrahydrofuran (THF) furnished compound **3**. The starting material was treated with methanesulfonyl chloride (MsCl) in pyridine in the presence of NaOH and dimethyl sulfoxide (DMSO) to yield a mixture of compound **4** and the monosubstituted product. Compound **4** was finally purified by silica gel column chromatography, and the monosubstituted product was converted to the epoxide (compound **5)** by treatment with sodium ethoxide in THF. The synthesis of all the above compounds are shown in Scheme 1 and discussed below and all reactions are carried out according to the literature protocols [19,20,21].

### 2.2. Antifouling Assay

**Figure 1:**
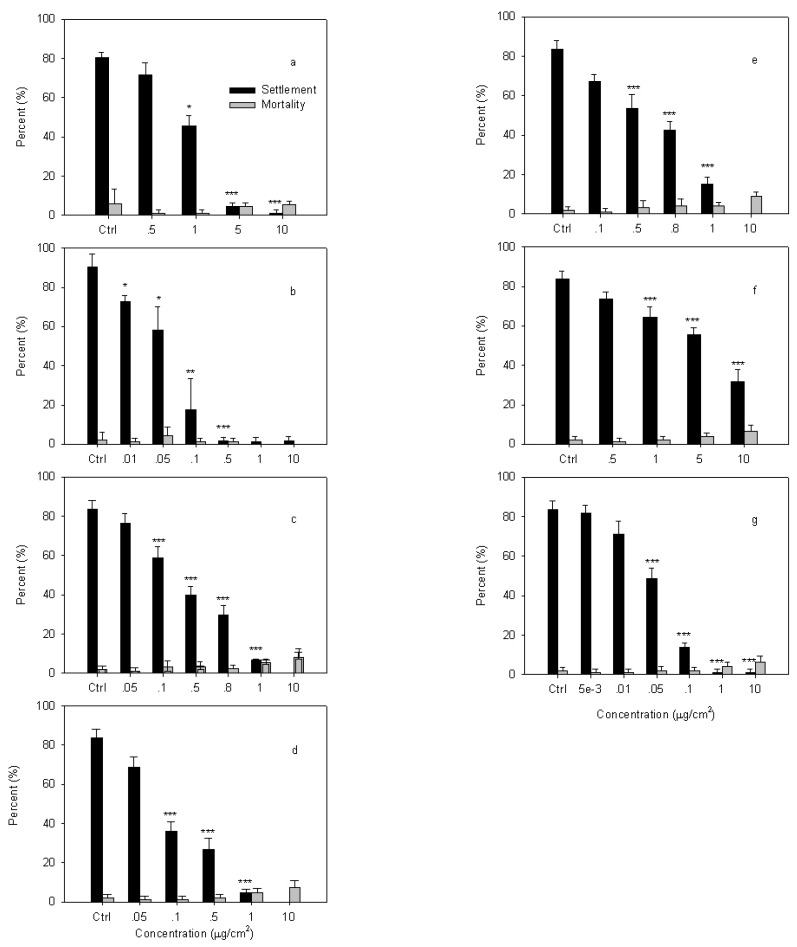
Effects of the compounds on settlement and survival of *B. albicostatus* cyprids. a: Capsaicin; b: ent-8(14)-Pimarene-15*R*,16-diol (starting material); c: 15s,-16-di-*O*-Acetyl-ent-8(14)-pimarane (**1**); d: 16-*O*-Acetyl-ent-8(14)-pimarane-15*S*-ol (**2**); e: 15*S*,-16-Dimethoxy-ent-8(14)-pimarane (**3**); f:15*S*,-16-di-*O*-Methanesulfonyl-ent-8(14)-pimarane (**4**); g: 15*S*,-16-ent-Epoxypimarane (**5**). Data were analyzed using one-way ANOVA, where *P < 0.05; **P < 0.01 and ***P < 0.001 were significantly different from the control.

The effects of the synthesized compounds on settlement and mortality of *B. albicostatus* cyprids are shown in Figure 1 and their EC_50_ and LC_50 _values are summarized in Table 1. Capsaicin, with an EC_50_ value of 1.32 µg/cm^2^, was used as a positive standard (Figure 1 and Table 1). As shown in Figure 1, all of the compounds, with EC_50_ values ranging from 0.04 to 7.47 µg/cm^2^, significantly inhibited settlement compared with the control (*P* < 0.001) without significant toxicity (*P* > 0.05). Capsaicin significantly affected larval settlement at 5 µg/cm^2^ (*P* < 0.001), and the starting material significantly affected larval settlement at 0.5 µg/cm^2^ (*P* < 0.001). Moreover, compounds **1-5** significantly reduced larval settlement at 0.1-1 µg/cm^2^ (*P* < 0.001). Compounds **1, 2, 3, 5** were more active than capsaicin, while compound **4** showed an antifouling activity weaker than capsaicin. Furthermore, the non-toxicity compounds were defined as those that do not directly kill fouling organisms at or near the levels at which they deter fouling [22]. 

**Table 1:** Antifouling activity of the investigated compounds against *B. albicostatus* cyprid larvae.

Compound	Compound name	EC_50_ (µg/cm^2^)	LC_50_ (µg/cm^2^)
Positive control	Capsaicin	1.32 ± 0.02	> 10
Starting material	ent-8(14)-Pimarene-15*R*,16-diol	0.04 ± 0.00	> 10
Compound **1**	15s,-16-di-*O*-Acetyl-ent-8(14)-pimarane	0.30 ± 0.01	> 10
Compound **2**	16-*O*-Acetyl-ent-8(14)-pimarane-15*S*-ol	0.14 ± 0.01	> 10
Compound **3**	15*S*,-16-Dimethoxy-ent-8(14)-pimarane	0.57 ± 0.01	> 10
Compound **4**	15*S*,-16-di-*O*-Methanesulfonyl-ent-8(14)-pimarane	7.47 ± 0.13	> 10
Compound **5**	15*S*,-16-ent-Epoxypimarane	0.05 ± 0.00	> 10

The LC_50_ values of these compounds were all greater than their EC_50_, indicating that they all inhibited cyprid settlement in a non-toxic way. In addition, stability tests showed that over a 2-month exposure under FSW compounds **1, 2, 3, 4** and starting material were degraded, while compound **5** remained stable.

Our SAR analysis of these pimarane diterpenoids demonstrated that the substitution patterns at the C-15 and C-16 position of pimarane diterpenoid significantly affected their antifouling activity. The starting material, which possesses hydroxyl groups at the C-15 and C-16 positions, led to the most potent antifouling activity. When other substituents, such as the acetoxy, methoxy, or a methanesulfonyloxy group were introduced on the C-15 and C-16 of the pimarane diterpenoid, the corresponding compounds showed the less potent activity than the starting material. Moreover, the antifouling activity of the methylated compound **3 **was lower than the acyl compounds **1** and **2**, suggesting that the acetoxy group at the suitable position of pimarane diterpenoid might result in a more potent antifouling activity than the methoxy group. On the other hand, compound **4** showed weak antifouling activity when compared to the positive control and other pimarane diterpenoids, suggesting that the methanesulfonyloxy group has no significant impact on the antifouling activity of primarane diterpenoids. In addition, when the hydroxyl groups at the C-15 and C-16 position in pimarane diterpenoid were substituted by an epoxy group, the resulting compound (compound **5**) showed similar antifouling activity as the starting material. Moreover, compound **5** showed better stability than starting material after 2-month exposure in filtered seawater (FSW 0.22 µm, salinity 30‰, and temperature 25 °C). These results suggest that the epoxy group might be another important functional group expressing potent antifouling activity in pimarane diterpenoids. Furthermore, all of the compounds were devoid of toxicity indicating that the substituent in the side chain of the compounds did not influence the toxicity of pimarane diterpenoids.

## 3. Experimental

### 3.1. General

^1^H- and ^13^C-NMR spectra were measured with a Varian INOVA 600 spectrometer (USA). ESI mass spectra data were recorded on an AB 3200Q TRAP spectrometer (USA). 200-300 mesh Silica gel (Qingdao Marine Chemical Factory, Qingdao, China) were used for column chromatography. Reactions were monitored by TLC on GF 254 silica gel (Qingdao Marine Chemical Factory, Qingdao, China). Capsaicin with purity above 95% was purchased from Sigma Chemical Co. (USA). 

*15S,-16-di-O-Acetyl-ent-8(14)-pimarane* (**1**). The starting material (100 mg, 0.33 mmol) in anhydrous triethylamine (0.1 mL) was treated with acetic anhydride (0.023 mL, 0.24 mmol) at 0 °C. After stirring at room temperature for 10 h, the mixture was quenched after consumption of starting material (TLC monitoring). Then, the mixture was extracted with CH_2_Cl_2_ three times. The total extract was washed with saturated aqueous NaCl solution, and dried with Na_2_SO_4_. After evaporation, the residue was subjected to silica gel column chromatography eluting with petroleum ether-acetone (11:1) to obtain compound **1** as a colorless oil (37% yield). ESIMS *m/z*: 391.3 [M + 1]^+^; ^1^H-NMR (CDCl_3_): δ_H_ 5.18 (1H, dd, *J* = 2.5, 9.2 Hz, H-16), 5.14 (1H, s, H-14), 4.40 (1H, dd, *J* = 2.5, 11.8 Hz, H-15), 4.02 (1H, dd, *J* = 9.2, 11.7 Hz, H-16), 0.95 (3H, s, H-17), 0.87 (3H, s, H-18), 0.84 (3H, s, H-19), 0.81 (3H, s, H-20), 2.07 (3H, s, COCH_3_), 2.02 (3H, s, COCH_3_); ^13^C-NMR (CDCl_3_) δ_C_ 38.3 (C-1), 18.0 (C-2), 41.2 (C-3), 32.3 (C-4), 53.9 (C-5), 21.6 (C-6), 35.2 (C-7), 138.2 (C-8), 49.3 (C-9), 37.5 (C-10), 17.6 (C-11), 30.7 (C-12), 35.5 (C-13), 125.4 (C-14), 76.2 (C-15), 62.8 (C-16), 23.1 (C-17), 32.7 (C-18), 21.1 (C-19), 14.1 (C-20), 170.0 (C=O), 169.4 (C=O), 20.0 (CH_3_ of COCH_3_), 19.8 (CH_3_ of COCH_3_).

*16-O-Acetyl-ent-8(14)-pimarane-15S-ol* (**2**). Compound **2** was synthesized like compound **1** but isolated by chromatography (petroleum ether-acetone 13:1) as colorless oil (13% yield). ESIMS *m/z*: 349.5 [M + 1]^+^; ^1^H-NMR (CDCl_3_): δ_H _5.18 (1H, s, H-14), 4.16 (1H, dd, *J* = 2.5, 9.5 Hz, H-16), 3.89 (1H, dd, *J* = 2.5, 10.5 Hz, H-15), 3.62 (1H, dd, *J* = 9.5, 10.5 Hz, H-16), 0.82 (3H, s, H-17), 0.76 (3H, s, H-18), 0.72 (3H, s, H-19), 0.66 (3H, s, H-20), 1.98 (3H, s, COCH_3_); ^13^C-NMR (CDCl_3_) δ_C _39.2 (C-1), 19.0 (C-2), 42.1 (C-3), 33.3 (C-4), 54.9 (C-5), 22.7 (C-6), 36.3 (C-7), 139.9 (C-8), 50.3 (C-9), 38.5 (C-10), 18.8 (C-11), 32.1 (C-12), 37.3 (C-13), 126.9 (C-14), 76.2 (C-15), 66.5 (C-16), 23.4 (C-17), 33.8 (C-18), 22.1 (C-19), 15.1 (C-20), 21.1 (CH_3_ of COCH_3_), 171.4 (C=O).

*15S,-16-Dimethoxy-ent-8(14)-pimarane* (**3**). The starting material (50 mg, 0.16 mmol) in THF (1.5 mL) was treated with sodium hydride (9.32 mg, 0.39 mmol), and the reaction mixture was stirred at room temperature for 3 h. Then, methyl iodide (0.02 mL, 0.32 mmol) was added into the mixture with continuous stirring at room temperature for 8 h. After that, the mixture was poured into saturated aqueous NH_4_Cl solution and then extracted with ethyl acetate three times. The total extract was washed with saturated aqueous NaCl solution, dried with Na_2_SO_4 _and evaporated. The residue was chromatographed over silica gel (elution with 25:1 petroleum ether- acetone) yielded compound **3** as a colorless oil (93% yield). ESIMS *m/z*: 357.4 [M + Na]^+^; ^1^H-NMR (CDCl_3_): δ_H_ 5.26 (1H, s, H-14), 3.49 (1H, dd, *J* = 2.6, 10.3 Hz, H-16), 3.31 (1H, dd, *J* = 7.3, 10.3 Hz, H-16), 3.12 (1H, dd, *J* = 2.6, 7.3 Hz, H-15), 0.81 (3H, s, H-17), 0.80 (3H, s, H-18), 0.77 (3H, s, H-19), 0.74 (3H, s, H-20), 3.39 (3H, s,OCH_3_), 3.28 (3H, s, OCH_3_); ^13^C-NMR (CDCl_3_): δ_C _38.2 (C-1), 18.0 (C-2), 41.2 (C-3), 32.3 (C-4), 53.9 (C-5), 21.8 (C-6), 35.2 (C-7), 135.9 (C-8), 49.4 (C-9), 37.6 (C-10), 17.7 (C-11), 30.9 (C-12), 36.5 (C-13), 128.3 (C-14), 86.1 (C-15), 73.5 (C-16), 22.5 (C-17), 32.7 (C-18), 21.1 (C-19), 14.1 (C-20), 59.5 (OCH_3_), 58.0 (OCH_3_).

*15S-16-di-o-Methanesulfonyl-ent-8(14)-pimarane* (**4**). The starting material (50 mg, 0.16 mmol) in anhydrous pyridine (0.2 mL) was treated with methanesulfonyl chloride (0.015 mL, 0.19 mmol), and the reaction mixture was stirred at 0 °C for 10min. Then, a solution of NaOH (19.6 mg, 0.49 mmol) in water (0.2 mL) plus DMSO (0.13 mL) was added into the mixture over 5 min with continuous stirring at 0 °C for 10 min. After this time, the mixture was poured into ice-water and then extracted with aether. The total extract was washed with saturated aqueous NaCl solution, dried with Na_2_SO_4 _and evaporated. Column chromatography on silica gel (petroleum ether-acetone 10:1) gave the monosubstitution production (20% yield), and column chromatography on silica gel (petroleum ether-acetone 20:1) gave compound **4** as a white powder (37% yield). ESIMS *m/z*: 485.3 [M + Na]^+^; ^1^H-NMR (CDCl_3_): δ_H _5.22 (1H, s, H-14), 4.86 (1H, dd, *J* = 1.7, 8.6 Hz, H-16), 4.50 (1H, dd, *J* = 1.9, 11.5 Hz, H-15), 4.32 (1H, dd, *J* = 8.6, 11.3 Hz, H-16), 1.01 (3H, s, H-17), 0.88 (3H, s, H-18), 0.84 (3H, s, H-19), 0.81 (3H, s, H-20), 3.09 (3H, s, SO_2_CH_3_), 3.09 (3H, s, SO_2_CH_3_);^13^C-NMR (CDCl_3_): δ_C _38.2 (C-1), 17.9 (C-2), 41.0 (C-3), 32.3 (C-4), 53.8 (C-5), 21.5 (C-6), 36.9 (C-7), 139.7 (C-8), 49.2 (C-9), 38.2 (C-10), 17.5 (C-11), 30.6 (C-12), 37.6 (C-13), 125.0 (C-14), 85.3 (C-15), 67.0 (C-16), 23.1 (C-17), 32.7 (C-18), 21.1 (C-19), 14.0 (C-20), 36.2 (SO_2_CH_3_), 35.0 (SO_2_CH_3_).

*15S,-16-ent-Epoxypimarane* (**5**). The monosubstitution production (40 mg, 0.13 mmol) in THF (1.5 mL) was treated with sodium ethoxide (6.8 mg, 0.1 mmol), and the reaction mixture was stirred at room temperature for 90 min. The mixture was poured into ice-water, then extracted with saturated aqueous NH_4_Cl solution three times, and extracted with aether three times. The total extract was washed with saturated aqueous NaCl solution, dried with Na_2_SO_4 _and evaporated. Column chromatography on silica gel (petroleum ether-acetone 60:1) gave compound **5** as colorless oil (36% yield). ESIMS *m/z*: 289.8 [M + 1]^+^; ^1^H-NMR (CDCl_3_): δ_H _5.03 (1H, s, H-14), 2.79 (1H, dd, *J* = 2.8, 9 Hz, H-16), 2.56 (1H, dd, *J* = 4.5, 9 Hz, H-15), 2.51 (1H, dd, *J* = 2.8, 4.5 Hz, H-16), 0.88 (3H, s, H-17), 0.87 (3H, s, H-18), 0.85 (3H, s, H-19), 0.80 (3H, s, H-20); ^13^C-NMR (CDCl_3_): δ_C _41.2 (C-1), 18.2 (C-2), 41.5 (C-3), 33.2 (C-4), 53.8 (C-5), 21.5 (C-6), 35.0 (C-7), 139.3 (C-8), 49.7 (C-9), 38.5 (C-10), 18.0 (C-11), 32.7 (C-12), 37.1 (C-13), 123.9 (C-14), 32.3 (C-15), 57.8 (C-16), 24.0 (C-17), 33.4 (C-18), 21.1 (C-19), 13.7 (C-20).

### 3.2. Antifouling Assay

*B. albicostatus* adults were collected from the intertidal zone in Xiamen, China. Adults released the stage I and II nauplius upon immersion in seawater after drying for 12 h [10]. Naupilar larvae actively swimming towards the light were collected using a pipette. They were cultured in FSW by feeding with the diatom *Chaetoceros muelleri* at a concentration of 2.0 × 10^5^ cells·mL^-1^. Each day seawater and diet were renewed. After 5~6 days, larvae reached the cyprid stage [10], and then the cyprids were collected within 24 h. Antifouling efficacies of the five synthetic diterpenoids were investigated according to the method of Hellio *et al*. with a minor modification [23]. Tested compounds were dissolved in CH_2_Cl_2_, and aliquots of the solution were added to the glass dishes and air-dried. To each glass dish were added 30 cyprids and 10 mL FSW. There were three replicates for the FSW control and each of the compound concentrations. The dishes were incubated in the dark at 25 °C for 48 h. After that, the numbers of larvae which settled, died or did not settle in each replicate were enumerated under a stereomicroscope. In addition, the stability of diterpenoid was demonstrated by means of their 2-month exposure in FSW. Each diterpene was applied to the dish, and then FSW was poured on the dish. Three replicates were set up for each of the experiment. After 2-month exposure in FSW, these compounds were dissolved in CH_2_Cl_2_, which was then evaporated. By comparison of these compounds, no differences were found in weight before and after 2-month exposure in FSW. These indicated that all of compounds were difficult to be dissolved in the FSW, and these compounds might be bound to the surface of the petri dish. Furthermore, the stabilities of these compounds were tested using the method of TLC, which were identified by comparison of the spots and Rf value of these compounds before and after 2-month exposure in FSW. Compound showing the same spots and Rf value as those before were regarded as stable. Compounds that showed the different spots and Rf values from those before were regarded as degraded. One-way ANOVA followed by a Dunnett's test for multiple comparisons of treatment means with a control, was used for the settlement or mortality percentages analysis. The significance level was set as 5%. The EC_50_ (the concentration that reduces the settlement rate by 50% relative to the control) and LC_50_ (the concentration that results in 50% mortality relative to the control) both with a 95% confidence interval were estimated using the Spearman-Karber method [24,25].

## 4. Conclusions

Five new pimarane diterpenoids were synthesized and evaluated against settlement of cyprid larvae of the barnacle *B. albicostatus.* These diterpenoids, which significantly reduced larval settlement at 0.1-1 µg/cm^2^ (*P* < 0.001), exhibited barnacle antifouling activities with EC_50_ values ranging from 0.04 to 7.47 µg/cm^2^. Compound **5**, which exhibited almost the same antifouling activity as the starting material, showed better stability than the latter, indicating that compound **5** has better commercial potential than the starting material. Moreover, the SAR demonstrated that the functional groups on the C-15 and C-16 position of pimarane diterpenoid were responsible for the antifouling activity.
